# Upregulation of DGCR8, a Candidate Predisposing to Schizophrenia in Han Chinese, Contributes to Phenotypic Deficits and Neuronal Migration Delay

**DOI:** 10.3389/fpsyt.2022.873873

**Published:** 2022-04-15

**Authors:** Yan Bi, Shiqing Chen, Qi Shen, Zhenming Guo, Decheng Ren, Fan Yuan, Weibo Niu, Lei Ji, Liangjie Liu, Ke Han, Tao Yu, Fengping Yang, Xi Wu, Lu Wang, Xingwang Li, Shunying Yu, Yifeng Xu, Lin He, Yi Shi, Jing Zhang, Weidong Li, Guang He

**Affiliations:** ^1^Bio-X Institutes, Key Laboratory for the Genetics of Developmental and Neuropsychiatric Disorders, Shanghai Jiao Tong University, Shanghai, China; ^2^Shanghai Key Laboratory of Psychotic Disorders, Brain Science and Technology Research Center, Shanghai Jiao Tong University, Shanghai, China; ^3^Institute of Neuroscience, State Key Laboratory of Neuroscience, CAS Center for Excellence in Brain Science and Intelligence Technology, Chinese Academy of Sciences, Shanghai, China

**Keywords:** DGCR8, schizophrenia, rs3757, neuronal migration, behavioral phenotype

## Abstract

DiGeorge Syndrome Critical Region Gene 8 (DGCR8) is a key component of the microprocessor complex governing the maturation of most microRNAs, some of which participate in schizophrenia and neural development. Previous studies have found that the 22q11.2 locus, containing DGCR8, confers a risk of schizophrenia. However, the role of DGCR8 in schizophrenia and the early stage of neural development has remained unknown. In the present study, we try to identify the role of DGCR8 in schizophrenia from human samples and animal models. We found that the G allele and GG genotype of rs3757 in DGCR8 conferred a higher risk of schizophrenia, which likely resulted from higher expression of DGCR8 according to our test of dual-luciferase reporter system. Employed overexpression model *in utero* and adult mice, we also revealed that the aberrant increase of Dgcr8 delayed neuronal migration during embryological development and consequently triggered abnormal behaviors in adult mice. Together, these results demonstrate that DGCR8 may play a role in the etiology of schizophrenia through regulating neural development.

## Introduction

Schizophrenia (MIM181500) is a debilitating mental illness mainly characterized by hallucinations, delusions, dampened emotions, social withdrawal and cognitive deficits (1). The global age-standardized point prevalence of schizophrenia is estimated to be 0.28% (95% uncertainty interval: 0.24–0.31) (2). It has been established that individuals with 22q11.2 microdeletions, removing a series of genes including *DGCR8*, exhibit a spectrum of cognitive deficits and approximately 30% of them develop typical schizophrenia or schizoaffective disorder in adolescence or early adulthood (3), which account for up to 1–2% of sporadic schizophrenia cases (4). *DGCR8*, one of the best-known genes disrupted by the 22q11.2 microdeletion (5, 6), is essential to microRNA production by encoding an RNA-binding moiety of the “microprocessor” complex (7, 8).

Animal model *Df(16)A^±^* mice, carrying a hemizygous 1.3-Mb chromosomal deficiency syntenic to the 1.5-Mb human 22q11.2 microdeletion, have consistently shown abnormalities in sensory information processing, synaptic connectivity in the hippocampus, working memory and migration of interneurons (5, 6, 9, 10), which are also seen in schizophrenia patients. Strikingly, analysis of *Dgcr8*^±^ strain provided compelling evidence that *Dgcr8* haploinsufficiency and the ensuing anomalies in miRNA biogenesis account, at least in part, for the neuronal disruption and abnormal behavior observed in *Df(16)A^±^* mice (5, 11). While the link of schizophrenia with 22q11.2 deletions as well as *DGCR8* depletion has been well-established in the animal study, there are few functional studies of microduplications of *DGCR8*/22q11.2 in schizophrenia (12–14).

Human and mouse tissues from adult and fetus showed rather ubiquitous expression of DGCR8/Dgcr8 (15), including subventricular zone/ventricular zone (SVZ/VZ), cortical plate (CP) and hippocampus (11, 16). The essential functions of Dgcr8 during development of the central nervous system involved in neuronal progenitor cells (NPCs) expansion, corticogenesis at early stages of development (16, 17) and neurogenesis in adult hippocampus (11). Previous study reported that deletion of Dgcr8 in postmitotic neurons of the postnatal mouse hippocampus and neocortex was reported to induce lethal phenotype (18). Overexpression of DGCR8 in the embryonic mouse neocortex promotes the expansion of NPC pools and represses neurogenesis (19).

Moreover, increased miRNA biogenesis and DGCR8, an RNA binding protein intimately involved in miRNA processing, were observed in the schizophrenia cohort (20) and it was featured in postmortem brain study that DGCR8 was significantly upregulated in the superior temporal gyrus (STG), the dorsolateral prefrontal cortex (DLPFC) (12) and hippocampus (21) which was in line with the elevation of schizophrenia-associated miRNA.

Thus, we hypothesized that *DGCR8* may be partially responsible for schizophrenia and abnormal expression of DGCR8 may result in developmental deficits in the neural system and abnormal behavioral phenotype in adulthood.

## Materials and Methods

### Subjects

In this case-control study, a total of 1,034 unrelated schizophrenia patients (625 males and 409 females, age: 52.1 ± 12.9 years old) and 1,034 unrelated healthy controls (588 males and 446 females, age: 41.1 ± 10.0 years old) were recruited. All subjects were Han Chinese in origin. All of the patients were chronic schizophrenia recruited from Shanghai Mental Health Center (Mental Health Center affiliated to Shanghai Jiao Tong University School of Medicine), Shanghai Changning Mental Health Center and Wuhu No.4 People’s Hospital (Wuhu psychiatric hospital) from 2001 to 2003. Schizophrenia cases were diagnosed by two independent psychiatrists using DSM-IV (Diagnostic and Statistical Manual of Mental Disorders – Fourth Edition) criteria. All the control subjects underwent a personal interview with a 10-item questionnaire based on the Mini-International Neuropsychiatric Interview (version 5.0.0), and none was found to have any history of psychiatric illness. The study was approved by the Ethics Committee of the Human Genetics Center in Shanghai. Written informed consent was obtained from all the individuals tested.

### Animal

Timed pregnant C57BL/6 10-week-old female mice were purchased from SLAC Laboratory Animal for *in utero* gene transfer and C57BL/6 8-week-old male mice for behavioral tests. Each five mice were housed in one cage, given food and water and maintained on a 12 h light, 12 h dark cycle. Animal care procedures were approved by the Institutional Animal Care and Use Committee of Shanghai Jiao Tong University.

### Antibodies

The primary antibodies used were: polyclonal anti-Dgcr8 (Proteintech), mouse anti-β-tubulin (Sigma), rabbit anti-β-actin (CST).

The secondary antibodies used were: HRP-anti-rabbit (Kang Chen), Alexa fluor 488 anti-rabbit (Invitrogen).

### Genotyping

Genomic DNA was extracted from peripheral blood samples of the subjects using the phenol-chloroform method. All SNPs were genotyped on the ABI 7900 DNA detection system (Applied Biosystems, Foster City, CA, United States) using TaqMan^®^ technology. All probes were designed by the Applied Biosystems service. The standard 5ml Polymerase chain reaction (PCR) was carried out using TaqMan^®^ Universal PCR Master Mix reagent kits under the guidelines provided.

### Plasmid Construction

Luciferase plasmids were constructed as follows. Primers were designed to amplify the 3′ UTR of *DGCR8* in humans with tags introducing the *Xba*I restriction enzyme site: 5′-GCTCTAGAgggaggtggcacggg (Forward) and 5′-GCTCTAGAttcccttctggacagtttgctttatg (Reverse). After amplification, PCR product was digested by *Xba*I. The 3′ UTR fragment was then inserted into the *Xba*I site in a pGL3-promoter firefly luciferase reporter vector (Promega) (pGL3-promoter-DGG); after which pGL3-promoter-DGA was created using Quickchange Lightning Site-directed Mutagenesis Kit (Stratagene). The primers for mutation were 5′-ctgatgccatccagagcactgggctgtgc (F) and 5′ gcacagcccagtgctctggatggcatcag (R). Both constructs were sequence verified prior to use.

Full-length mouse *Dgcr8* cDNA was cloned from cerebral cortical cDNA and inserted into pCAGIG overexpression vector by *Eco*RI and *Not*I to make pCAGIG-*Dgcr8* for *in utero* gene transfer. And full-length mouse *Dgcr8* cDNA was inserted into pCMV-mcherry for lentivirus package.

### Luciferase Assay

Human embryonic kidney 293 (HEK293) cells were cultured in Dulbecco’s modified Eagle’s medium with 10% fetal bovine serum, 100 U/ml penicillin, 100 U/ml streptomycin at 5% CO2 and 37°C. At 80–90% confluence in a 24-well culture dish, the cells were transiently cotransfected with pRL-SV40 Renilla reporter vector (Promega) and pGL3-promoter-DGA or pGL3-promoter-DGG or pGL3-promoter using FuGENE HD Transfection Reagent (Roche) according to the manufacturer’s protocol. Twenty-four hours after transfection, the cells were harvested and the activities of both the firefly luciferase and Renilla luciferase were measured using the Dual-Luciferase Reporter Assay System (Promega) on Berthold LB940 according to the manufacturer’s protocols. Renilla luciferase values were used to normalize cell counts and transfection efficiency.

### Secondary Structure Prediction

Secondary structures of 3′ UTR of *DGCR8* with rs3757 polymorphism were predicted using the CentroidFold program available at http://www.ncrna.org/centroidfold and the RNAfold program available at http://rna.tbi.univie.ac.at/cgi-bin/RNAWebSuite/RNAfold.cgi.

### Verification of Overexpression Efficiency of Constructs

HEK293 cells were transiently cotransfected with pCAG-GFP-Dgcr8 and shRNA vectors using Lipofectamine 2000 (Invitrogen) according to the manufacturer’s protocol. E18 primary cortical neurons were cultured as described by Zhou et al. (22). shRNAs were transfected into neurons using the Amaxa Rat Neuron Nucleofector Kit and the Nucleofecor II Device (Lonza).

### *In utero* Electroporation

The *in utero* electroporation was carried out as described by Zhao et al. (23). Pregnant mice at E13.5 were anesthetized with 1% Nembutal solution (100 mg/kg, sigma). When the uterus was taken out, 1–2 μl plasmids and Fast Green (0.001%, sigma) mixture was microinjected into the lateral ventricle of embryos using a fine glass micropipette. The brains of embryos were then clamped by ECM830 (BTX Instrument Division, Harvard Apparatus Inc., Holliston, MA, United States) at five times of 30 V for 50 ms, with an interval of 100 ms. After injection, the uterine horn was placed back in the abdominal cavity and the abdominal wall and skin was surgically sutured. After surgery, the animal was placed in a warm recovery incubator for 1 h. A total of 12 mice were underwent the embryonic electroporation surgery.

### Virus Injection

Dentate gyrus (DG) was chosen for the target site of lentivirus infection where neurogenesis in the brain of adult mammals occurs throughout life. Mice were anesthetized with 1% Nembutal solution (100 mg/kg, sigma) and placed in a stereotactic apparatus. A small craniotomy was performed and 1 μl of lentivirus was gradually injected using a finely pulled capillary connected to a pulse generator and a vacuum pump. The following stereotactic coordinates were used relative to Bregma for DG: caudal 2.0, lateral 1.6, and ventral 1.9–2.1. A total of 32 mice were injected with the virus, that was 16 for lenti-Ctrl group and 16 for lenti-*Dgcr8* group.

### Western Blotting

Tissue or cells were lysed in RIPA buffer (Sigma) including a complete/phosphatase stop cocktail (Millipore). Protein concentration was measured by Bradford assay (Bio-rad) and lysates (20 μg per lane) were separated by 6–10% SDS-PAGE and transferred onto a nitrocellulose membrane. The transferred membrane was incubated with a blocking solution [5% milk (BD) in TBST] 1 h at room temperature, followed by overnight incubation with a primary antibody at 4°C. The membrane was incubated with HRP-conjugated secondary antibody (Millipore) for 1 h at room temperature. Immunoreactivity was detected with an ECL kit (Millipore). The optical density of the immunoreactivity bands was analyzed using ImageJ software (NIH). All western blotting experiments used a sample size of 3 versus 3.

### Slice Imaging and Analysis

The pattern of migration in the developing cerebral cortex, the numbers of GFP- positive cells was counted from four to five different littermates. Brain slices were obtained under a Leica confocal microscope. Cortical sublayers were identified based on cell density using nuclei dye or specific markers.

### Mouse Behavioral Tests

Due to the accidental death of 2 mice in control (lenti-Ctrl) group during the virus injection, eventually 30 mice (14 for lenti-Ctrl versus 16 for lenti-*Dgcr8*) entered the behavioral testing stage. Mice were measured in a novel open field (27.5 cm × 27.5 cm) for 20 min. The elevated plus maze was conducted in a device (two open arms and two closed arms with walls of 17 cm height, each arm is 29 cm long, and 8 cm wide.). In the sociability test, mice were allowed to explore in a three-chambered box with openings between the chambers for 10 min. After that, a strange little mouse was placed inside a wire containment cup that was located in one of the side chambers. Then the original mouse was replaced in the same box for 5 min. In the forced swim test, mice were placed in an 18 cm diameter and 25 cm high container and recorded for 5 min. The context fear conditioning test was carried out by using operant chambers (Med Associates) with electrifiable steel grid floor. During the training phase, mice were habituated to the chamber for 2 min, followed by 3 times foot shock. 24 h after the training, the mice were placed in the same chamber for 5 min to record their reaction. The Startle Response System of SR – LAB™ company was applied for prepulse inhibition test (PPI). Mice were in the soundproof box with a pressure sensor to 5 min, and then tested 90 trials. The background noise was set as 65 decibels, strongly stimulated as 120 decibels, former pulse as 76, 79, or 85 decibels. The apparatus for Morris water maze test consists of a circular water tank filled with water and a hidden platform submerged a few centimeters under the water surface in one quadrant of the tank. Training of the mice to locate the hidden platform take for 7 days and tested spatial learning of these mice at the 8th day where the platform was removed from the pool.

### Statistical Analysis

Genotypic association and Hardy-Weinberg equilibrium were calculated on analysis.bio-x.cn (24), a powerful software platform with integrated analysis tools appropriate for association studies. 100,000 permutations of positive genotypes were performed using gplink (version 2.050). Linkage disequilibrium (LD) and allele frequencies were analyzed using Haploview 4.0RC1 (25). 100,000 further permutations of positive alleles were also obtained using Haploview 4.0RC1. Haplotypic frequencies were initially estimated using UNPHASED including all window sizes and all marker combinations (26). 100,000 further permutations of positive haplotypes obtained from UNPHASED were corrected using Haploview 4.0RC1. The power of the SNP data in the case-control study was calculated using G*Power 3.1.2. The effect size was set at 0.1 (corresponding to a weak gene effect), and the significance level was set at 0.05.

The effect of polymorphisms of rs3557 on expression levels was tested by one-way analysis of variance (ANOVA) using GraphPad Prism 5. Values are presented as mean ± SEM. Western blot band intensities were qualified using Image Lab 3.0 (BIORAD). Neuronal distribution was analyzed by Image-Pro Plus 6.0. Values are presented as the mean ± SEM. Student’s *t*-test was used to measure the significance of differences between two groups, and one-way analysis of variance (ANOVA) was used for three or more groups.

## Results

### rs3757, a SNP in 3′ UTR of *DGCR8*, Is Associated With Schizophrenia in the Han Chinese Population

The abnormality of *Dgcr8*^±^ mice and strong association between 22q11.2 microdeletion and schizophrenia prompted us to speculate that DGCR8 expression levels could affect susceptibility to schizophrenia. In addition, many other studies have inferred that 3′UTR of a gene can significantly influence abundance of corresponding protein through regulating mRNA stability (27). We, therefore, chose 5 SNPs (rs1640299, rs2286928, rs417309, rs720012, and rs3757) from 3′ UTR of *DGCR8* to conduct an association study in 1034 schizophrenia patients and 1,034 healthy controls in the Han Chinese population. Among these five SNPs, rs1640299 and rs720012 are tag SNPs in the Chinese population.

Of these five SNPs, four met Hardy-Weinberg equilibrium in both patients and controls and rs720012 was the exception. Rs720012 was therefore discarded from further analysis. The statistical power of all four SNPs was above 90%. The allelic and genotypic frequencies of the four SNPs are listed in Table 1. Among the four SNPs, rs3757 showed a statistically significant difference in allelic and genotypic distribution between the schizophrenia group and the healthy group (*P* = 0.003 for allele; *P* = 0.008 for genotype). Both allelic and genotypic association remained after Bonferroni correction (*P* = 0.012 and 0.032, respectively). As higher frequencies were found in the patient group than in the control group, the G allele and GG genotype at rs3757 were probably conferred a higher risk of schizophrenia. In addition, we did gender stratification analysis, rs2286928 in male and rs3757 in female showed a statistically difference in allelic distribution between the schizophrenia group and the healthy group after Bonferroni correction (both *P* = 0.028).

**TABLE 1:** The distribution of alleles and genotypes for the four SNPs in DGCR8 3′ UTR.

SNP ID	Allele frequency		*p-*value^a^	genotypic frequency			*p-*value^a^	H-W *p*-value
rs1640299		A		AA	AC	CC		
	case	1528(0.766)	0.524	584(0.585)	364(0.364)	51(0.051)	1.000	0.554
	control	1496(0.746)		557(0.553)	389(0.386)	62(0.062)		0.587
rs2286928		A		AA	AG	GG		
	case	113(0.056)	0.320	6(0.006)	112(0.110)	896(0.884)	0.236	0.227
	control	90(0.044)		3(0.003)	84(0.083)	931(0.915)		0.453
rs417309		A		AA	AG	GG		
	case	76(0.039)	0.836	4(0.004)	72(0.074)	899(0.922)	0.840	0.055
	control	63(0.032)		1(0.001)	61(0.061)	932(0.938)		0.999
rs3757		G		AA	AG	GG		
	case	1566(0.825)	**0.012**	34(0.036)	267(0.280)	654(0.685)	**0.032**	0.301
	control	1535(0.787)		44(0.045)	330(0.337)	605(0.618)		0.906

*^a^p-values (after Bonferroni correction) <0.05 are in boldface.*

There was no strong linkage disequilibrium among the SNPs (Supplementary Figure 1). Haplotype analysis revealed that there were a series of positive associations of 2 phase, 3 phase, and 4 phase haplotypes with schizophrenia when the global *p*-value was below 0.05 (Table 2).

**TABLE 2:** Estimated haplotype frequencies in the case-control subjects.

	Test markers^a^	Haplotype	Ca-Freq	Co-Freq	*P*-value^a^	*P*-value^b^	Global *P*-value^c^
2 SNP	rs1640299-rs2286928	C-A	**0.05**	0.037	0.047	0.103	**0.017**
		C-G	0.183	0.217	0.0094	**0.015**	
	rs1640299-rs3757	C-A	0.146	0.183	0.003	**0.0065**	**0.015**
	rs2286928-rs417309	G-G	0.904	0.925	0.016	0.069	0.119
	rs2286928-rs3757	G-A	0.173	0.21	0.0038	**0.0066**	**0.021**
	rs417309-rs3757	G-A	0.172	0.211	0.0029	**0.0063**	**0.011**
		G-G	0.788	0.756	0.02	**0.046**	
3 SNP	rs1640299-rs2286928-rs417309	C-G-G	0.183	0.215	0.013	**0.023**	0.16
	rs1640299-rs2286928-rs3757	C-G-A	0.147	0.183	0.0031	**0.0045**	0.071
	rs1640299-rs417309-rs3757	C-G-A	0.144	0.183	0.0023	**0.0057**	**0.022**
	rs2286928-rs417309-rs3757	G-G-A	0.173	0.209	0.0037	**0.0083**	**0.013**
4 SNP	rs1640299-rs2286928-rs417309-rs3757	C-G-G-A	0.145	0.183	0.0025	**0.0032**	0.164

*Ca-Freq: the marginal frequency of the haplotypes in cases. Co-Freq: the marginal frequency of the haplotypes among controls. ^a^haplotypes with a p-value (before permutation) >0.05 are not shown. ^b^p-values(after permutation) <0.05 are in boldface. ^c^global p-values < 0.05 are in boldface.*

### rs3757 Polymorphism Affects Expression *in vitro*

Our association study identified rs3757 in *DGCR8* gene as a vulnerable factor to schizophrenia. The next question was whether this association had anything to do with the protein expression levels. To investigate this, we engineered luciferase reporters carrying either wild-type or mutated versions of *Dgcr8* 3′UTR. Two constructs containing A or G at rs3757 (pGL3-promoter-DGA, pGL3-promoter-DGG) were generated through site directed mutation and were transfected into HEK293 cells.

**FIGURE 1:**
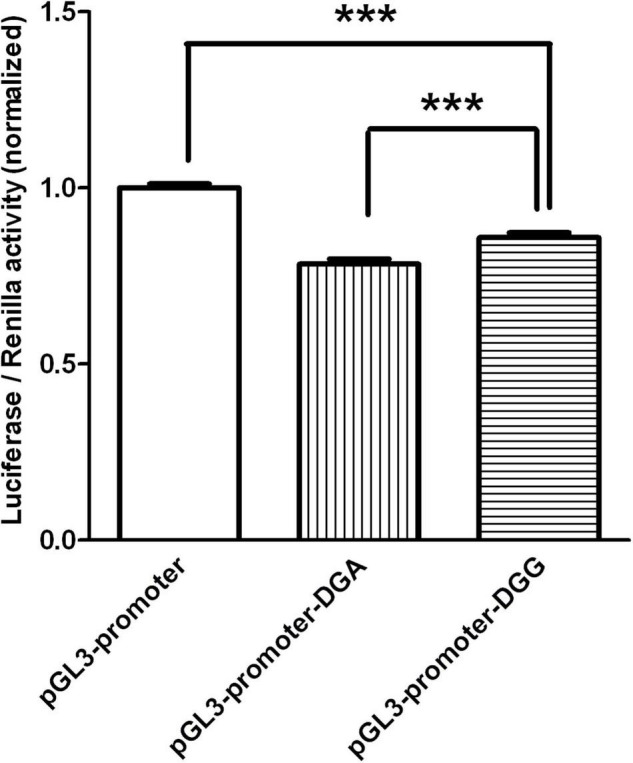
The luciferase activity of the two constructs transfected in HEK293 were graphed after normalization against the plasmid pRL-SV40 expressing the Renilla luciferase gene. Data of pGL3-promoter was presented as mean with SEM of *n* = 9 samples. Data of pGL3-promoter-DGA and pGL3-promoter-DGG were presented as means with SEM of *n* = 27 samples respectively. ***p < 0.001, One-way ANOVA test.

As shown in Figure 1, the luciferase activity of pGL3-promoter-DGG (DGG in short) was about 10% higher than that of pGL3-promoter-DGA (DGA in short), while the luciferase activities of both constructs were significantly lower than that of pGL3-promoter vector with no fragment inserted.

### Dgcr8 Overexpression Results in Abnormal Behaviors

**FIGURE 2:**
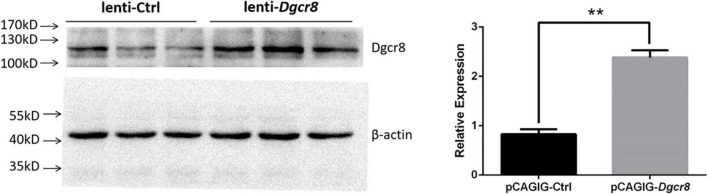
Dgcr8 was overexpressed in hippocampus of case group. Lenti-Ctrl or lenti-*Dgcr8* was injected into dentate gyrus of hippocampus. Two weeks later, extracts from hippocampus were immunoblotted with Dgcr8 antibody (ProteinTech Group, 10996-1-AP). Protein levels were normalized to β-actin, presented as means ± SEM. *N* = 3 for lenti-Ctrl and lenti-*Dgcr8*, respectively. One-way ANOVA test. **p = 0.0035.

Since the overexpression of Dgcr8 associated with human schizophrenia, we asked whether enhancing the expression of Dgcr8 would affect mice behaviors and cognition. We injected lentivirus lenti-*Dgcr8* (case group) or lenti-Ctrl (control group) into the DG region of the hippocampus in 8-week-old mice, and the location of viral injection was shown in Supplementary Figure 2. The efficiency of the lenti-*Dgcr8* was verified on protein levels (Figure 2). Two weeks after viral injection, we analyzed the behavioral and cognitive performance of two groups’ mice using various tests, including open field test, elevated plus maze test, social interaction test, forced swim test, fear conditioning test, PPI, and Morris water maze test.

**FIGURE 3:**
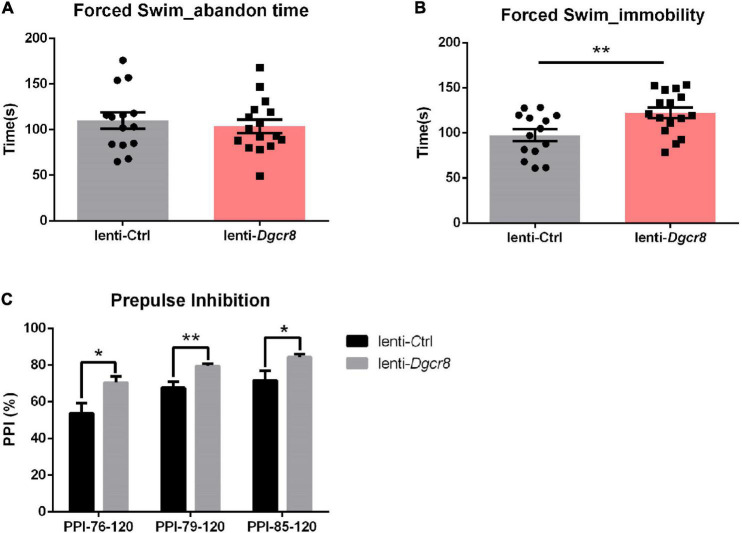
Results of the forced swim test. **(A)** Abandon time showed no significant difference between two groups (*p* = 0.5854). Abandon time: the time point when the mice initially gave up struggling in the water. **(B)** The case group showed more immobility than the control group (*p* = 0.0092). Immobility: the total amount of time the mice floated in the water during the 5 min testing. *N* = 14 for lenti-Ctrl group and *n* = 16 for lenti-*Dgcr8* group. Results were presented as means ± SEM. Owe-way ANOVA test. **p < 0.01. **(C)** Results of the prepulse inhibition (PPI) test. The prepulse stimulus (76, 79, and 85 dB) and the startle stimulus (120 dB) were used. The case showed significant higher PPI than the control group for each prepulse. *N* = 14 for lenti-Ctrl group and *n* = 16 for lenti-*Dgcr8* group. Results are presented as means ± SEM. Two-way ANOVA test. *p < 0.05, **p < 0.01.

We applied the forced-swim test to evaluate depression- and anhedonia-like behavior in lenti-*Dgcr8* mice, and used “abandon time” (the time point when the mice initially gave up struggling in the water) and “immobility time” (the total amount of time the mice floated in the water during the 5 min testing) to do the statistical results. It showed significantly increased immobility among lenti-*Dgcr8* micecompared with lenti-Ctrl mice (Figure 3B) and this abnormality also appeared in *Dgcr8*^±^ mice (11), suggesting abnormalities in *Dgcr8* gene trigger depression phenotype. Performance in PPI task, a measure of preattentive processing and a non-specific endophenotype of several psychiatric, displayed abnormal sensorimotor gating in lenti-*Dgcr8* mice (Figure 3C). Notably, several genes including Dgcr8 disrupted by 22q11.2 microdeletion have been pointed toward to modulate PPI repeatedly (5, 28–30). In addition, all the other behavior tests (open field, elevated plus maze, social interaction, fear conditioning and Morris water maze test) displayed negative results which were shown in Supplementary Figure 4.

### Dgcr8 Affects Neuronal Migration

**FIGURE 4:**
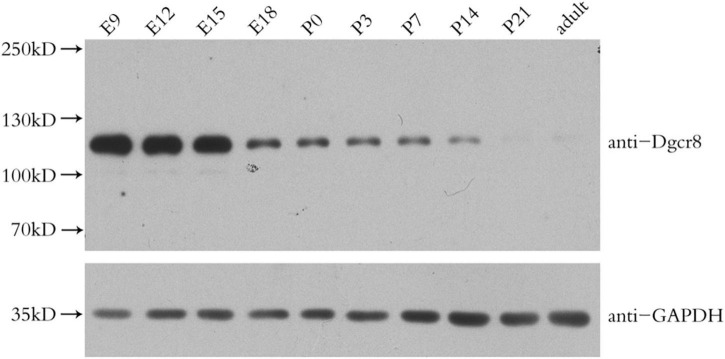
Western blot analysis of Dgcr8 protein levels in mice neocortex during cortical development at the indicated time points, including embryonic day (E) 9, 12, 15, 18, and postnatal day (P) 0, 3, 7, 14, 21 as well as adult age. *N* = 3 for each time point.

We found that Dgcr8 was highly expressed in the mice neocortex in the early stages of embryonic development and that it decreased gradually with the growth of embryos. At postnatal day 21 and beyond, Dgcr8 abundance was much lower, compared to that of E9 (Figure 4). In addition, increasing evidence has provided support for the neurodevelopmental hypothesis of schizophrenia (31). Previous studies had inferred that the miRNA pool changed dramatically during the first stage of development (32, 33) and that *Dgcr8* knockout mice died before birth. We therefore further investigated the role of *Dgcr8* in neural development.

We introduced Dgcr8 overexpression vectors into progenitors of the embryonic cerebral cortex using *in utero* electroporation to investigate the role of *Dgcr8* in the developing neocortex. The efficiency of the overexpression vector was verified in both the cell line and mouse cerebral cortex (Supplementary Figure 3).

**FIGURE 5:**
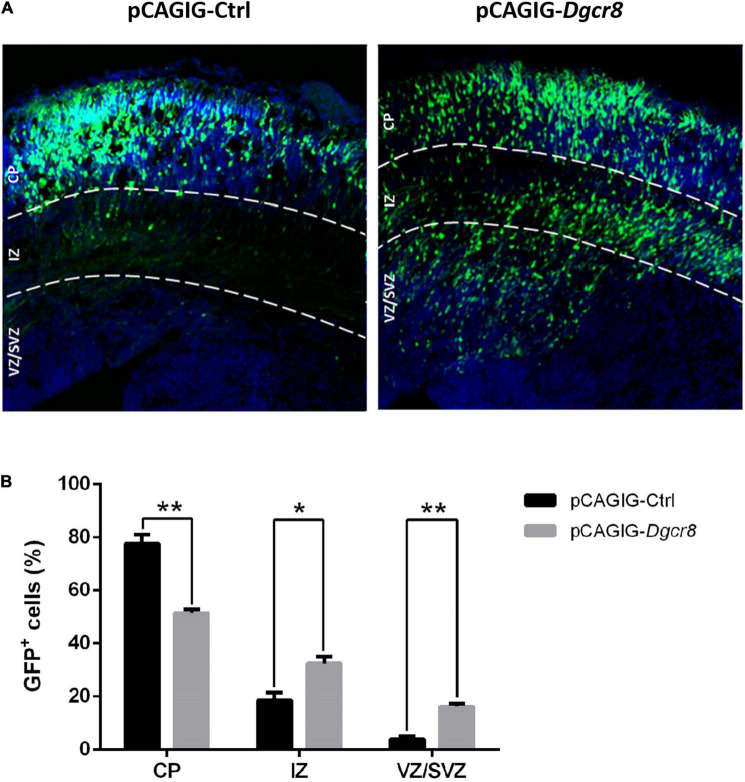
Overexpression of Dgcr8 in ceretral cortex at E13.5 delayed neural migration. Transfected cells expressed GFP, and sections were divided into three zones according DAPI staining (Blue), including VZ/SVZ (ventricular zone/subventricular zone), IZ (intermediate zone), and CP (cortical plate). At 16.5, the amount of GFP^+^ cells in every zone was counted and analyzed. Datas are presented as means ± SEM. Two-way ANOVA test. *p < 0.05; **p < 0.01.

pCAGIG-Ctrl and pCAGIG-*Dgcr8* were introduced into NPCs in the ventricular zone of the mouse cortex at embryonic day 13.5 (E13.5). The time point of *in utero* electroporation was determined by compromising between Dgcr8 expression profile in developing the mouse neocortex and feasibility of surgery. At embryonic day 16.5 (E16.5), we evaluated the distribution of EGFP-positive cells in the dorsolateral area of the neocortex in coronal sections (Figures 5A,B). In the pCAGIG-Ctrl group, and most neurons expressing EGFP (77.68 ± 5.70%) were located at the cortical plate at E16.5. However, when pCAGIG-*Dgcr8* was transfected into NPCs, the migration process of these transfected neurons was significantly delayed. At E16.5 and the percentage of neurons positioned at CP was much lower (51.40 ± 2.54%). Moreover, a considerable amount of EGFP positive neurons had not departed from the ventricular zone (VZ) and the subventricular zone (SVZ) (32.43 ± 4.38% for VZ and 16.17 ± 2.11% for SVZ) in the pCAGIG-*Dgcr8* mice. Thus, *Dgcr8* might play a role in normal neuronal migration.

## Discussion

22q11.2 deletion syndrome (22q11DS) constitutes one of the strongest genetic risks for schizophrenia. Many genes located in this region have been found to be associated with the disease (34, 35). However, these candidate genes do not fully explain symptoms observed in schizophrenia patients with 22q11.2 microdeletion, such as miRNA abnormal expression profiles in patients compared to healthy individuals. This infers that there may be other genes in the 22q11.2 microdeletion region responsible for specific symptoms. In the case-control study, we revealed the link between rs3757 in *DGCR8’s* 3 UTR and schizophrenia which is consistent with previous study from Zhou et al. in which rs3757 displayed a significantly increased risk with an odds ratio (OR) of 3.73 loci in 256 schizophrenia patients compared with 252 healthy controls of Chinese origin (36). In addition, only two groups have explored the relationship between *DGCR8* and schizophrenia prior to the present study (35, 37), while neither of these was based on Asian populations. Liu et al. genotyped two SNPs in *DGCR8* (rs2073778 in the promoter and rs1640299 in 3′ UTR) in South African and US samples using a family based study and a case-control study (35). They found no association, which was consistent with our finding that rs1640299 conferred no susceptibility to schizophrenia in the Chinese population. Another work analyzed three SNPs in *DGCR8* (rs2531721, rs374225, and rs2073778) among schizophrenia patients of Ashkenazi Jewish origin and the results still failed to reach statistical significance (37). Our investigation is, therefore, the first to report the correlation between *DGCR8* and schizophrenia based on genetic factors in relative large Chinese population (1,034 schizophrenia patients versus 1,034 health control). And according to our subsequent reporter gene test, the increased expression of *DGCR8* derived from rs3757 polymorphisms likely to initially explain this association.

It has been reported that 3′ UTR polymorphisms could affect the stability of mRNA as well as protein expression levels by altering the mRNA secondary structure (38, 39). We tried to predict a similar role for the secondary structures of 3′ UTR in *DGCR8* carrying A or G at rs3757 using CentroidFold (Supplementary Figure 5). This prediction inferred that 3′ UTR of *DGCR8* with G at rs3757 would have lower free energy than that with A at rs3757, which meant the former was more stable than the latter. Another RNA secondary structure prediction program – RNAfold replicated the difference. Concordant with the reporter gene assay showing that the luciferase activity of DGG was higher than that of DGA, it can be presumed that the polymorphism of rs3757 confers susceptibility to schizophrenia by regulating DGCR8 expression through changing the stability of *DGCR8* transcript. Indeed, evidence is showing that aberrant expression of DGCR8 was demonstrated in schizophrenia patients although the results are incongruence. Ota et al. (40) discovered that DGCR8 was downregulated in first episode of psychosis compared to people of health control and clinical high risk for psychosis while the study from Danielle et al. announced increased expression of DGCR8 and a broad increase in miRNA expression in schizophrenia (20). Nevertheless, more immediate evidence from postmortem cerebral samples of individuals with schizophrenia showed DGCR8 overexpressed in several region, including hippocampus (21) superior temporal cortex and dorsolateral prefrontal cortex (12), which strongly supported our results.

Unlike 22q11.2 microdeletion syndrome, whose mouse model has been among the few animal models that replicate abnormalities associated with schizophrenia, 22q11.2 microduplication syndrome has been detected relatively less (41) due to the lack of highly sensitive detection technology (12) or misdiagnosis (13), although a recent study suggested considerable frequency of this new syndrome (42). Despite highlighted diversity of symptoms in patients with 22q11.2 microduplication syndrome, many shared symptoms, such as abnormal behavior, cognitive defect, and cardiovascular malformations have also been found in patients with 22q11.2 microdeletion syndrome (42). In addition, Itsara et al. identified the link of 22q11.2 microduplication and schizophrenia using their own data and other published genome-wide data on schizophrenia, autism and mental retardation (43). Thus, it is possible that some genes located in 22q11.2, such as *DGCR8*, may undergo rigid regulation and any divergence from normal psychological expression level of these genes could lead to severe consequences.

In the forced-swim test, lenti-*Dgcr8* mice exhibited increased immobility compared with littermate control suggesting the depression-like behavior, an important characteristic feature of the negative symptoms of schizophrenia. Surprisingly, this increase in immobility time also appeared in Dgcr8-deficient mouse model (11). It is likely that the dysregulation of Dgcr8 could give rise to the depression phenotype and the underlying mechanism needs more refined work to uncover. Prepulse inhibition, which humans and rodents are tested in similar fashions, is a neural filtering process that allows attention to be focused on a given stimulus and is deficient in patients with schizophrenia. Several genes in 22q11.2 locus have been mapped as modulators for PPI, such as *Comt* (44), *Prodh* (28, 44), *Tbx1* (30), *Dgcr8* (5). These prior studies mentioned above consistently demonstrated lower PPI in heterozygous deficient mice compared to control, the abnormality seen in patients with schizophrenia. Only one report, however, is available as yet demonstrating increased PPI in mice with a deletion of the DGCR syntenic region including seven genes but not *Dgcr8* (29). Interestingly, we found Dgcr8-overexpression mice displayed significantly higher sensorimotor gating. To some extent, our results are agreed with precious work that discovered the Dgcr8^±^ mice showing reduced PPI. One explanation might be that DGCR8 has a dosage-sensitive effect on pathways important for PPI. And while PPI serves as a useful endophenotype for schizophrenia, modifications of the PPI are very diverse and could be caused by alteration of the voice in Parkinson’s disease subjects (45), high trait anxiety (46), short sleep duration in postpartum women (47), behavioral states (such as emotional context or a stressor) (48–50), hormonal levels (51). Even for schizophrenia, the lack of PPI attenuation has not been seen in all patients. Pioneer studies have shown the deficiency of PPI occurs only when the patient is in an acute symptomatic stage, or when antipsychotics are not being used (52). Furthermore, some also proposed that no statistically significant correlations between PPI and neuropsychological deficit in chronic schizophrenia was found in their study (53). Another possible factor that might contribute to higher PPI result in the present study is the mouse model. There are several strains of mice that develop high frequency hearing loss as they mature, like DBA/2 and C57BL/6, which makes them potentially unable to hear the prestimulus in late life (54). The mouse model in the present was generated by virus injection which may not be able to fully reflect the syndrome of schizophrenia. Besides, virus injection in mice may has a certain influence on sensorimotor gating, which may does not agree with schizophrenia phenotype.

What is the underlying mechanism of increased *Dgcr8* causing these abnormal behaviors? We tried to combine clinical results and behavior in mice. Our further exploration showed *Dgcr8* was highly expressed in the mice neocortex in the early stages of embryonic development and aberrant upregulation of Dgcr8 was involved in the delay of neuronal migration. NJ Beveridge et al. detected a substantial schizophrenia-associated increase in a host of microRNA expression from the post-mortem brain tissue of schizophrenia sufferers, which correlated with an ascent of pre-miRNA but not pri-miRNA processing and consistent with an augment in the microprocessor component *DGCR8* (12). miRNAs emerge as primary factors in the regulation of specific neurobiological functions containing neuronal migration, differentiation as well as synaptic plasticity. In particular, miR-107, among the most significantly upregulated miRNA in the study mentioned above (12), also implicated in delaying neuronal migration by targeting *CDK5R1* gene (55). Besides, overexpression of miR-9 could lead to the acceleration of neuronal migration through a feedback regulatory loop with a nuclear receptor TLX (56). Notably, current evidence from the pathway analysis demonstrated that the genes targeted by the alterant schizophrenia-associated microRNA highly enriched in neural connectivity and synaptic plasticity pathways, such as axon guidance, long-term potentiation, WNT, ErbB and MAP kinase signaling (12) and this corresponds with the deficits of *Dgcr8* mice (5). Collectively, these experiments were broadly shed light on the role of *Dgcr8* in the regulation of miRNA-associated pathway contributing to the pathophysiology of schizophrenia.

In summary, the present study showed for the first time that rs3757 at 3′ UTR of *DGCR8* was associated with schizophrenia via upregulating the expression of *DGCR8* in the Chinese Han population. Furthermore, elevating expression of Dgcr8 in mice delayed neuronal migration in the cerebral cortex and may consequently progress to abnormal behaviors in adulthood. The clinical findings along with data derived from animal approaches in the present study suggested that *DGCR8* dysregulation has a certain degree of influence on neurodevelopment. However, there are two main limitations of the present study need to declare. One is that the link between abnormal neuronal migration at early development stage and psychiatric behavior at adulthood stage due to the overexpression of Dgcr8 did not be confirmed. Another one is that the miRNAs which might be affected by the high expression of Dgcr8 were not detected. Further refined studies, extended to *DGCR8*-associated microRNAs and related pathways to confirm the underlying molecular mechanisms involved in the development of schizophrenia, are clearly warranted.

## Data Availability Statement

The original contributions presented in the study are included in the article/Supplementary Material, further inquiries can be directed to the corresponding authors.

## Ethics Statement

The studies involving human participants were reviewed and approved by the Ethics Committee of the Human Genetics Center in Shanghai. The patients/participants provided their written informed consent to participate in this study. The animal study was reviewed and approved by the Institutional Animal Care and Use Committee of Shanghai Jiao Tong University.

## Author Contributions

SC and JZ: conceptualization. YB and KH: data curation. GH: funding acquisition. LJ and LL: formal analysis. DR and FY: investigation. JZ and ZG: methodology. XW and LW: project administration. TY, XL, YX, SY, and FPY: resources. WL: supervision. LH: validation. YB: roles/writing – original draft. YS and YB: writing – review and editing, YB, QS, and WN: revising. All authors contributed to the article and approved the submitted version.

## Conflict of Interest

The authors declare that the research was conducted in the absence of any commercial or financial relationships that could be construed as a potential conflict of interest.

## Publisher’s Note

All claims expressed in this article are solely those of the authors and do not necessarily represent those of their affiliated organizations, or those of the publisher, the editors and the reviewers. Any product that may be evaluated in this article, or claim that may be made by its manufacturer, is not guaranteed or endorsed by the publisher.
