# Supplementary material for: Upregulation of DGCR8, a Candidate Predisposing to Schizophrenia in Han Chinese, Contributes to Phenotypic Deficits and Neuronal Migration Delay
Source: Front Psychiatry. 2022 Apr 15;13:873873. doi: 10.3389/fpsyt.2022.873873 (PMC9051063; doi:10.3389/fpsyt.2022.873873)
Supplement: Supplementary file 1 [file Data_Sheet_1.pdf]

Supplementary Material

Supplementary Table 1. Allele and genotype distribution in male or female for the schizophrenia patients and healthy controls

SNP ID			allele frequency	p value	genotypic frequency			p value	H-W p value
rs1640299			A		AA	AC	CC		
	male	case	920(0.759)	0.867	347(0.573)	226(0.373)	33(0.054)	0.974	0.629
		control	865(0.754)		324(0.566)	217(0.379)	31(0.054)		0.493
	female	case	612(0.779)	0.108	237(0.603)	138(0.351)	18(0.046)	0.082	0.712
		control	638(0.732)		233(0.534)	172(0.394)	31(0.071)		0.923
rs2286928			A		AA	AC	CC		
	male	case	86(0.070)	**0.028**	3(0.005)	80(0.130)	533(0.865)	0.056	0.992
		control	51(0.044)		3(0.005)	45(0.078)	530(0.917)		0.064
	female	case	38(0.048)	0.738	3(0.008)	32(0.080)	363(0.912)	0.175	0.021
		control	39(0.044)		0(0.000)	39(0.089)	401(0.911)		0.331
rs417309			A		AA	AC	CC		
	male	case	41(0.035)	0.876	1(0.002)	39(0.066)	550(0.932)	0.617	0.723
		control	38(0.034)		0(0.000)	38(0.067)	528(0.933)		0.408
	female	case	39(0.051)	0.104	3(0.008)	33(0.086)	349(0.906)	0.103	0.033
		control	25(0.029)		1(0.002)	23(0.054)	404(0.944)		0.279
rs3757			C		AA	AC	CC		
	male	case	208(0.182)	0.094	23(0.040)	162(0.284)	385(0.675)	0.188	0.258
		control	234(0.210)		19(0.034)	196(0.353)	341(0.613)		0.151
	female	case	127(0.165)	**0.028**	11(0.029)	105(0.273)	269(0.699)	0.108	0.845
		control	184(0.217)		25(0.059)	134(0.317)	264(0.624)		0.153

p-values after Bonferroni correction < 0.05 are in boldface

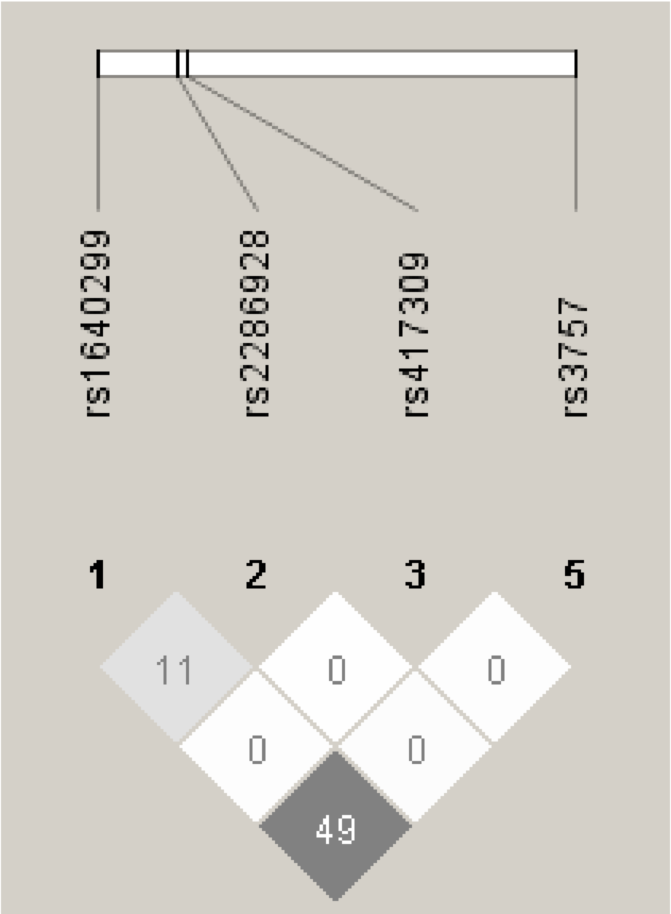

Supplementary Figure 1. LD between SNPs of DGCR8. r^2 between marker pairs is indicated by the shaded matrices.

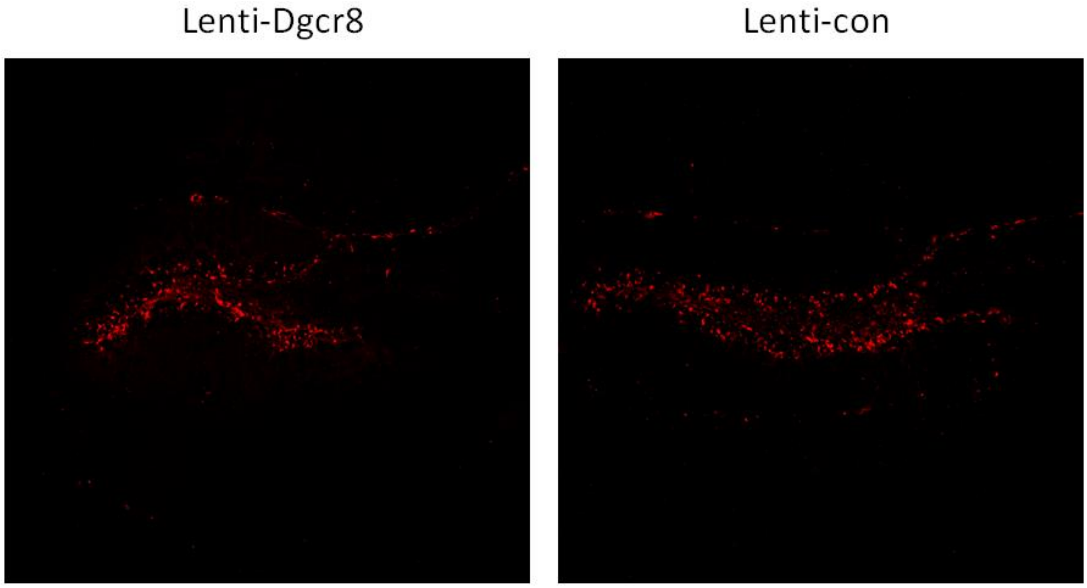

Supplementary Figure 2. Position of lentiviral injection. Both of lenti-Dgcr8 (left) and lenti-con (right) infected the dentate gyrus of hippocampus.

(A)

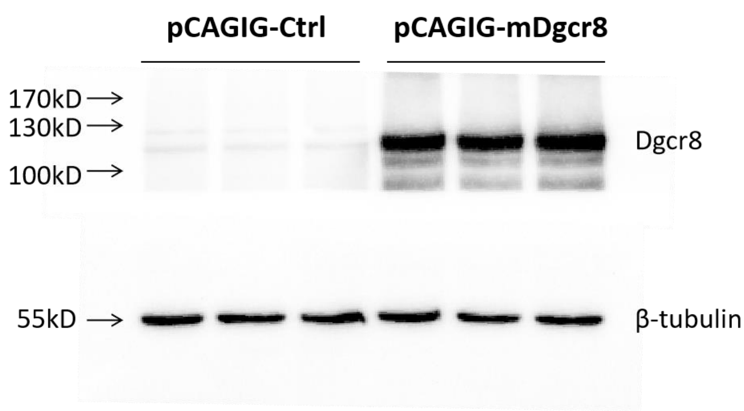

(B)

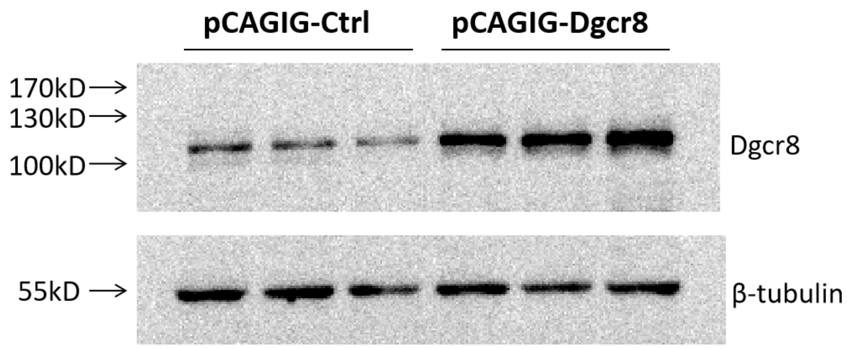

(C)

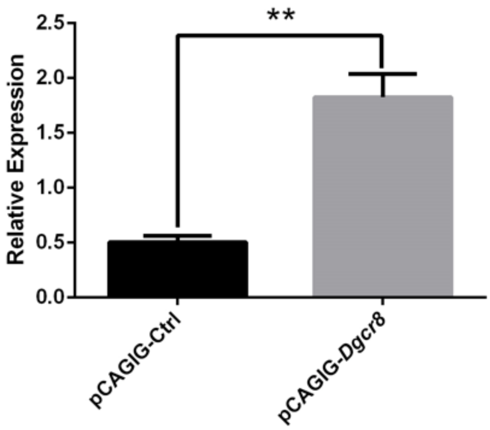

Supplementary Figure 3. The overexpression efficiency of constructs in 293T cells and cerebral cortex. (A). pCAGIG-Dgcr8-cDNA plasmid could overexpress Dgcr8 in 293T cells. Extracts from 293T cells transfected with pCAGIG-ctrl or pCAGIG-mDgcr8 plasmid were immunoblotted with Dgcr8 antibody (ProteinTech Group, 10996-1-AP). (B&C). Dgcr8 was overexpressed in cerebral cortex of case group. At E13.5, cerebral cortex of mouse embryo was transfected with pCAGIG-ctrl or

pCAGIG-mDgcr8 in utero electroporation. At 16.5, extracts from cerebral cortex were immunoblotted with Dgcr8 antibody (ProteinTech Group, 10996-1-AP). Protein levels were normalized to β-tubulin, presented as means ± SEM. One-way ANOVA test. $p=0.0039$.

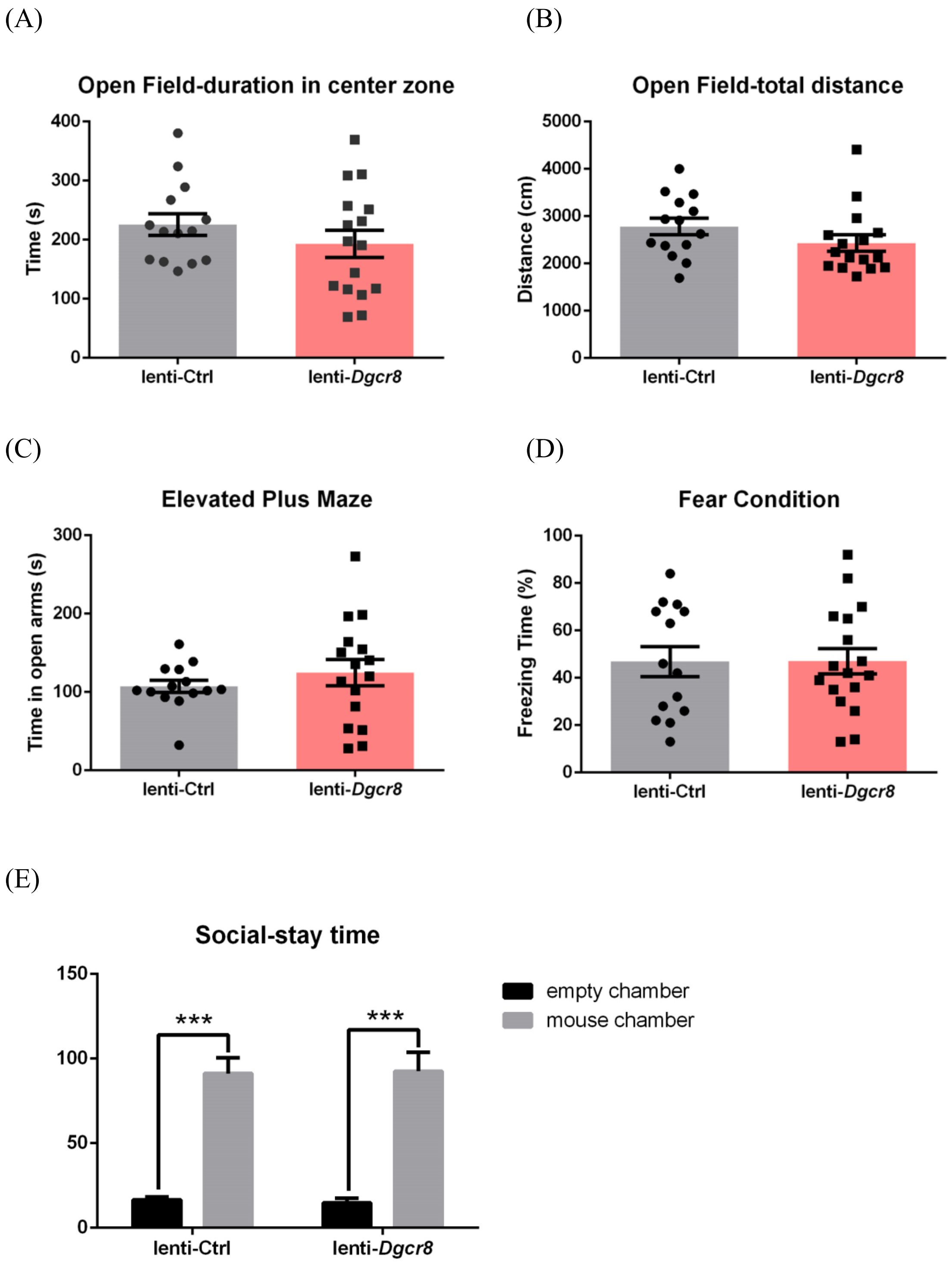
(A)
Open Field-duration in center zone
Time (s)
0
100
200
300
400
lenti-Ctrl
lenti-*Dgcr8*
(B)
Open Field-total distance
Distance (cm)
0
1000
2000
3000
4000
5000
lenti-Ctrl
lenti-*Dgcr8*
(C)
Elevated Plus Maze
Time in open arms (s)
0
100
200
300
lenti-Ctrl
lenti-*Dgcr8*
(D)
Fear Condition
Freezing Time (%)
0
20
40
60
80
100
lenti-Ctrl
lenti-*Dgcr8*
(E)
Social-stay time
0
50
100
150
***
***
lenti-Ctrl
lenti-*Dgcr8*
empty chamber
mouse chamber

(F)

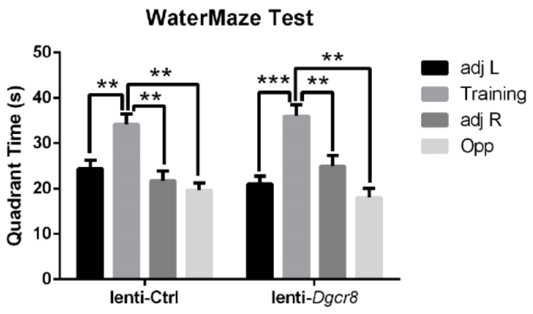

(G)

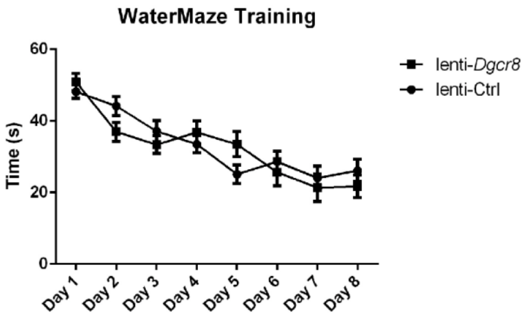

Supplementary Figure 4. Results of behavioral experiment. N=14 for lenti-Ctrl group and n=16 for lenti-*Dgcr8* group, mean ± SEM. (A & B) Open field: Student's t-test showed no significant difference in the total distance or duration in center zone between control (lenti-Ctrl) and Dgcr8 overexpression group (lenti-*Dgcr8*). (C) Elvated plus maze: Two-way ANOVA showed no significant difference in the time spent during the open arm region between lenti-Ctrl group and lenti-*Dgcr8* group. (D) Fear condition: Student's t-test showed no significant difference in the freezing time between lenti-Ctrl group and lenti-*Dgcr8* group. (E) Social ability test: Two-way ANOVA showed no significant difference between lenti-Ctrl group and lenti-*Dgcr8* group, and significant intragroup differences between mouse chamber and empty chamber (***P<0.001). (F) Water Maze Test: The proportion time spent in the four different quadrants of the mice in lenti-Ctrl group and lenti-*Dgcr8* group after 7-day training. Training represented the quadrant where the platform was located, adj L and adj R were the left and right quadrants of Training respectively, and Opp was the opposite quadrant of Training. (G) Water Maze Test: Seven-day learning curve showed no significant difference between lenti-Ctrl group and lenti-*Dgcr8* group.

(A) (B)

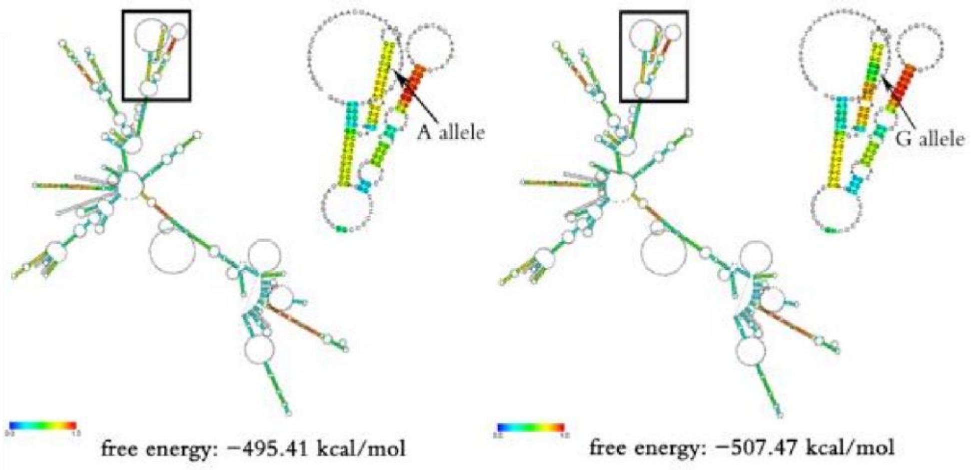

Supplementary Figure 5. The predicted secondary structure of DGCR8 3'UTR with A allele (A) or G allele (B) at rs3757 using CentroidFold with default parameters. Both structures are colored to indicate base-pairing probability. Arrows indicate the region in which rs3757 is located.
